# Paeoniflorin Attenuates Dexamethasone-Induced Apoptosis of Osteoblast Cells and Promotes Bone Formation via Regulating AKT/mTOR/Autophagy Signaling Pathway

**DOI:** 10.1155/2021/6623464

**Published:** 2021-04-07

**Authors:** Liyu Yang, Shengye Liu, Shuai Mu, Ran Guo, Long Zhou, Qin Fu

**Affiliations:** Department of Orthopedics, Shengjing Hospital of China Medical University, Shenyang, Liaoning 110003, China

## Abstract

Paeoniflorin, a natural product derived from *Paeonia lactiflora*, possesses diverse pharmacological activities such as anti-inflammatory, antitumor, and antidiabetic effects. It has been reported for promoting osteoblastogenesis and inhibiting osteoclastogenesis. This study investigates the therapeutic effects of paeoniflorin in glucocorticoid-induced osteoporosis (GIOP) *in vitro* and *in vivo*. MC3T3-E1 cells were incubated with dexamethasone (DEX; 200 *μ*M) and/or paeoniflorin (10 *μ*M), followed by the investigation of cell proliferation, differentiation, mineralization, apoptosis, and autophagy. The AKT activator SC79 was used for evaluating the involvement of the AKT/mTOR signaling pathway. After DEX pretreatments, paeoniflorin promoted osteoblast differentiation and mineralization characterized by increase in Runx2, ALP, beclin-1, and LC3-II/LC3-I ratio levels and a decrease in apoptosis. The autophagy-promoting effects of paeoniflorin were reversed by SC79. C57BL/6 mice were given DEX (1 mg/kg) once daily and paeoniflorin (15 mg/kg) 48 hours for a total of 8 weeks followed by the investigation of histological changes, the trabecular bone microarchitecture, and the levels of bone turnover markers. The results showed that paeoniflorin increased alkaline phosphatase (ALP) activity and upregulated the expression of osteocalcin and beclin-1 but reduced the levels of Bax and C-terminal telopeptide of type I collagen (CTX-1). Thus, paeoniflorin may alleviate DEX-induced osteoporosis by promoting osteogenic differentiation and autophagy via inhibition of the AKT/mTOR signaling pathway.

## 1. Introduction

Glucocorticoids (GCs) are widely used for amelioration of inflammation and treatment of inflammation-mediated different diseases due to their anti-infective and immunosuppressive effects. However, long-term treatment and high doses of GC commonly lead to various complications. Among these complications, GC-induced osteoporosis (GIOP) is the leading cause of secondary osteoporosis [1]. Clinical evidences show that GCs treatment of GIOP patients results in decline of bone mass and fracture risk [2]. A previous study has linked the pathogenesis of GIOP with reduced bone formation, thus indicating the destruction of bone homeostasis [3]. Though GCs have shown the suppression of osteoblast numbers and osteogenic differentiation activities, the exact mechanism underlying GIOP and its potential therapeutic targets are still unclear and require further investigation.

Exposure to dexamethasone (DEX) induces osteoblast apoptosis while inhibiting MC3T3-E1 cell proliferation [4]. Apoptosis involves three stages [5]. The fate of the cell is particularly determined in the second stage, known as the recognition phase, thus indicating the interaction between apoptotic and survival signals [5]. Through this cell signaling process, the metabolic status of osteoblasts is converted to apoptosis or autophagy for responding to the DEX-induced stress. An imbalance between apoptosis and autophagic activity may cause GIOP. Autophagy is an intracellular cleaning process that removes damaged organelles and combats harmful abnormalities [6]. Autophagy may help to prevent osteoblasts exposed to DEX stress from apoptosis by maintaining homeostasis [7, 8], indicating that the relationship and communication between autophagy and apoptosis are important in the pathogenic mechanism of GIOP. Therefore, the identification of a drug that can modulate the abnormalities in the apoptosis and autophagy of osteoblasts affected by DEX would be a new therapeutic target. Osteogenesis is the first step in the osteointegration of materials as osteoblasts are directly responsible for the differentiation and activity of their osteoclast counterparts. Osteoblast commitment and differentiation are controlled by complex activities. Many factors are involved in the regulation of osteoblastogenesis. Bone morphogenetic proteins and the Wnt glycoproteins play crucial roles in signaling osteoblast commitment and differentiation and are the only known factors capable of initiating osteoblastogenesis from uncommitted progenitors [9].

For the treatment of inflammatory and autoimmune disorders, osteoporosis is among the most damaging side effects of glucocorticoid (GC) therapy. Evidence from both humans and mice suggests deleterious skeletal consequences, both related and unrelated to a reduction in bone mineral density, within weeks of pharmacological GC administration (BMD). Osteoclast numbers and bone resorption are also rapidly increased, and these improvements contribute to the fastest loss of BMD during the initial disease process, along with osteoblast inactivation and reduced bone formation [1]. Bone resorption then decreases to subphysiological levels, but recurrent and extreme bone formation inhibition leads to more bone loss and a steadily increased risk of fracture, up to an order of magnitude greater than that seen in untreated individuals. Thus, the major culprits in GIOP are known to be bone-forming osteoblasts [1, 10].

Paeoniflorin, a monoterpene glucoside, is extracted from the root of *Paeonia lactiflora* Pall. Being an active Chinese herbal medicine ingredient, paeoniflorin is widely prescribed for its anti-inflammatory and diuretic effects [11]. Various other therapeutic applications including immunoregulation, antiallergic, antioxidant, and anticancer activities have been recently explored for paeoniflorin [12]. In terms of bone metabolism, paeoniflorin can suppress osteoclastogenesis and facilitate osteoblastogenesis by regulating the NF-*κ*B pathway [13]. It can also induce autophagy to prevent cell injury [14]. Accordingly, we estimated the effects of paeoniflorin on apoptosis and autophagy in MC3T3-E1 cells and C57BL/6 mice exposed to DEX.

## 2. Materials and Methods

### 2.1. Drugs and Reagents

Purified paeoniflorin and DEX (>98%) were purchased from Sigma-Aldrich (St. Louis, MO, USA). They were dissolved in dimethyl sulfoxide (DMSO; Sigma-Aldrich) to prepare their 100 mM stock solutions and were diluted in a cell culture medium for obtaining different interventional concentrations. Cell Counting Kit-8 (CCK-8) and Annexin V-FITC Apoptosis Detection Kit were obtained from Dojindo Molecular Technologies, Inc. (Rockville, MD, USA). Alkaline phosphatase (ALP) activity assay kit, ALP, and Alizarin Red staining reagents were obtained from Beyotime Institute of Biotechnology (Jiangsu, China). The phosphorylation-specific antibodies of AKT and mammalian target of rapamycin (mTOR), together with their pan-antibodies, were obtained from Cell Signaling Technology, Inc. (Danvers, MA, USA). Runx2, Bcl-2, Bax, cleaved caspase-3, and total caspase-3 antibodies were purchased from Abcam (Cambridge, MA, USA) for use in western blot and immunochemistry. SC79, an AKT activator, was dissolved in DMSO and diluted with cell culture medium to 1 *μ*M.

### 2.2. Cell Culture

The MC3T3-E1 osteoblastic cell was obtained from the Chinese Academy of Medical Sciences and the Institute of Basic Medical Sciences Cell Resource Center in Shanghai. MC3T3-E1 cells were cultured in *α*-MEM supplemented with 10% fetal bovine serum (Biological Industries, Israel). The culture medium was changed every other day and the cells were incubated at 37°C in a 5% CO_2_ containing incubator.

### 2.3. Cell Proliferation Assay

MC3T3-E1 cells were seeded at a density of 5 × 10^3^ cells/well in 96-well plates and observed to attach overnight. The DEX and paeoniflorin groups were treated with different concentrations of DEX and paeoniflorin alone for 24 h, whereas the DEX + paeoniflorin group was pretreated with various concentrations of paeoniflorin for 4 h followed by the addition of 200 *μ*M DEX for 24 h. After 24 h, 10 *μ*l of CCK-8 reagent was added for 2 h. Absorbance was measured in triplicate at 450 nm using a microplate reader. MC3T3-E1 and its derivatives are widely used tools in bone research, having been cited over 4000 times since their introduction in 1981. They express osteogenic gene expression, matrix deposition, and mineralization and can differentiate into mature osteoblasts [15].

### 2.4. Alkaline Phosphatase Activity/Staining Assay

For ALP staining assay, MC3T3-E1 cells were seeded at 2 × 10^5^ cells/well in 6-well plates. In the DEX + paeoniflorin group, the osteoblasts were pretreated with 10 and 20 *μ*M paeoniflorin for 4 h followed by their exposure to different concentrations of DEX for 24 hours. To determine ALP activity, the cells were lysed with 100 *μ*L of assay lysis buffer. An ALP reagent kit (Nanjing Jiancheng Bioengineering Research Institute, Nanjing, China) was used according to the manufacturer's instructions. A standard curve was plotted using p-nitrophenol as the standard, and the ALP activity of each sample was evaluated from the optical density (450 nm) values.

### 2.5. Alizarin Red Staining

MC3T3-E1 cells were incubated for 14 days in 6-well plates at a density of 1 × 10^3^ cells/well. After indicated interventions, cells were fixed with 4% paraformaldehyde for 20 minutes at room temperature followed by their staining with 0.1% Alizarin Red S (Beyotime Institute of Biotechnology) staining solution for 40 minutes. After rinsed thrice with PBS, the cells were photographed under the microscope.

### 2.6. Flow Cytometric Analysis of Osteoblast Apoptosis

Preosteoblast MC3T3-E1 cells were cultured at a density of 2 × 10^5^ cells/well in 6-well plates for 24 h followed by their treatment with DEX in the presence or absence of 10 or 20 *μ*M paeoniflorin for 24 h. The cells were harvested and resuspended in 500 *μ*L binding buffer containing 5 *μ*L Annexin V-FITC and 5 *μ*L propidium iodide. After rinsed twice with PBS, the cells were placed in an ice bath for 30 minutes and samples were analyzed by FACScan flow cytometry.

### 2.7. Hoechst Staining of Osteoblast Apoptosis

For Hoechst staining, 10 *μ*L Hoechst live cell staining solution (Beyotime Institute of Biotechnology) was added to the culture medium and mixed gently. Then MC3T3-E1 cells were incubated at 37°C for 10 min. The culture solution containing dye was aspirated and the cells were washed 2–3 times with PBS and observed under a fluorescence microscope. The nuclei of apoptotic cells were dense or fragmented.

### 2.8. Western Blot Analysis

Osteoblastic MC3T3-E1 cells were treated with or without paeoniflorin in 6-well plates for 2 days. After being washed three times in PBS, the cells were incubated in lysis buffer for 30 minutes on ice. Total protein concentrations were determined with a BCA protein assay kit (Beyotime Biotechnology). Equal protein amounts from each group were separated on 10% sodium dodecyl sulfate-polyacrylamide (SDS-PAGE) gels and electrophoretically transferred onto polyvinylidene difluoride (PVDF) membranes. The membranes were blocked for 2 h at room temperature using a 5% concentration of skim milk with Tris-buffered saline containing Tween-20 (TBST) solution. After being washed with TBST, the membranes were incubated overnight at 4°C with primary monoclonal antibodies against Bcl-2, Bax, Runx2, caspase-3, AKT, mTOR, beclin-1, and LC3. The membranes were then incubated for 2 h at room temperature with horseradish peroxidase-conjugated secondary antibody. Chemiluminescent signals were developed with enhanced chemiluminescent reagent (32109; Thermo Scientific, Waltham, MA, USA) and detected with a chemical gel documentation system.

### 2.9. Quantitative Real-Time Polymerase Chain Reaction Analysis of Autophagy-Related Genes

MC3T3-E1 cells were incubated for 2 days in 6-well plates, and total RNA was extracted by using TRIzol reagent (Takara Bio, Dalian, China). cDNA was synthesized from 1 *μ*g total RNA from each sample using a PrimeScript RT reagent kit with a gDNA eraser (Takara Bio). The cDNA was amplified with SYBR premix (PrimeScript RT Master Mix; Takara Bio). The specific primers applied for detecting the mRNA transcripts are shown in Table 1. Quantitative real-time polymerase chain reaction (qRT-PCR) was performed on an ABI 7500 detection system (Applied Biosystems, CA, USA). The PCR data were analyzed using ABI Prism 7000 SDS software and the relative levels of gene expression were determined with the ΔCq = Cq gene − Cq reference and calculated by the 2^−ΔΔCq^ method. Analyses were independently repeated in triplicate.

### 2.10. Animals and Drug Treatments

Twenty-four 8-week-old female C57BL/6 mice (weighing 18–20 g) were obtained from Hua-Fu-Kang Biotechnology Co. (Beijing, China). Mice were acclimatized for 1 week under pathogen-free laboratory conditions at the experiment center of Shengjing Hospital of China Medical University under controlled temperature and humidity conditions and a 12 h light/dark cycle with free access to food and water. All animal care and experimental procedures were approved by the Institutional Animal Care Ethics and Use Committee of China Medical University (protocol approval number, 2020PS382K).

Mice were evenly and randomly assigned to one of three groups: control group, DEX group, and DEX with paeoniflorin group. The GIOP model was established through intramuscular injection of 1 mg/kg/day DEX for 60 days. The control group was administered equivalent amounts of normal saline. The DEX + paeoniflorin group received 15 mg/kg/day paeoniflorin by intraperitoneal injection in conjunction with the same induction protocol applied to the GIOP model group. After 8-week treatments, mice were sacrificed to evaluate the change in bone metabolism and microstructure.

### 2.11. ELISA Detection of Bone Turnover Markers

Serum was collected from GIOP mice treated with paeoniflorin after centrifugation for 20 min at 12,000 rpm. OCN was measured and calculated using a commercially available Sandwich ELISA (enzyme-linked immunosorbent assay) kit (MK127; Takara Bio, Japan) and analyzed in accordance with the manufacturer's instructions. Serum CTX-1 was determined by using an AC-06F1 ELISA kit (Immunodiagnostic Systems, Tyne & Wear, UK).

### 2.12. Histological and Immunohistochemical Assays

Femurs were cleaned off tissue, fixed in 4% paraformaldehyde at 4°C for 24 h, and then decalcified in 10% ethylenediaminetetraacetic acid (pH 7.2) at room temperature for 14 days. The segmental bone was embedded in paraffin and cut into 3.5 *μ*m thick sections. Then, sections were stained with hematoxylin and eosin (H&E) (Solarbio Biotechnology, Beijing, China) for histological analysis. The sections were then deparaffinized and briefly washed with PBS for immunohistochemistry. They were then incubated in 3% H_2_O_2_ to block endogenous peroxidase activity. Bax and beclin-1 primary antibodies were incubated with bone sections overnight at 4°C. The sections were cleaned with PBS and incubated with goat anti-rabbit horseradish peroxidase-conjugated secondary antibodies for 30 min. The immunostained sections were then dyed with DAB. A series of five random fields was photographed per section. The areas of positive (immunoreactive) cells and total cells in each section were counted and quantitatively characterized using Image-Pro Plus 6.0.

### 2.13. Statistical Analysis

All experimental data were presented as the mean ± standard deviation of at least three independent experiments and were analyzed by using GraphPad Prism 6.0. One-way analysis of variance (ANOVA) was used to calculate the statistical difference. *P* values less than 0.5 were considered statistically significant. Immunochemistry was evaluated with Image-Pro Plus 6.0.

## 3. Results

### 3.1. The Pretreatment with Paeoniflorin Tends to Improve MC3T3-E1 Cell Viability, ALP Activity, and Mineralization in Cells Exposed to High Dose of DEX

**Figure 1:**
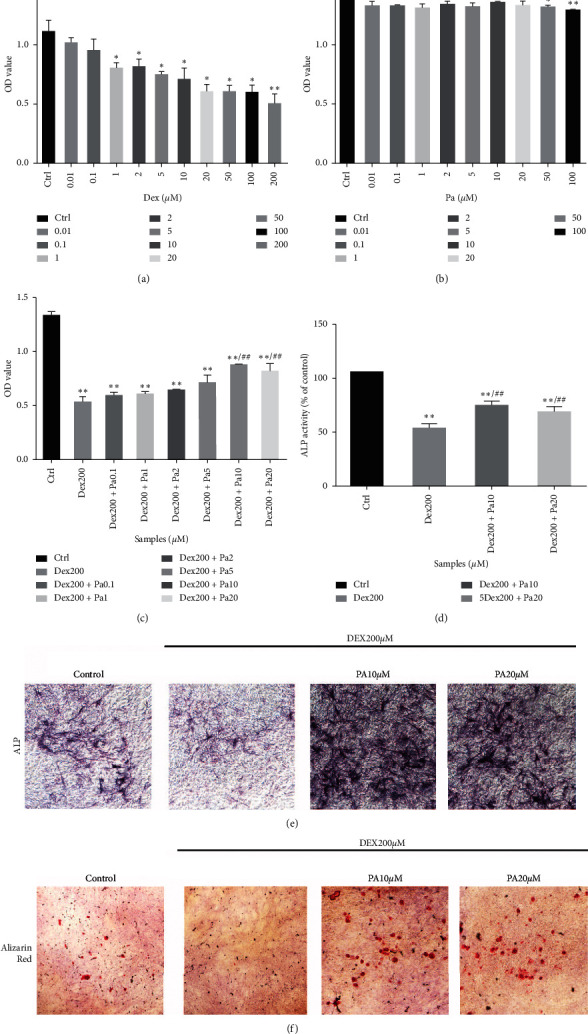
Effect of paeoniflorin on the viability and osteogenic differentiation of MC3T3-E1 induced by DEX (a, b). The cells were incubated with different concentrations of dexamethasone (DEX) and paeoniflorin (Pa) for 24 hours alone. Cell viability was determined by the Cell Counting Kit-8(CCK-8) assay. Measurements were in triplicate and data are presented as the mean ± SD.  ^*∗*^*P* < 0.05;  ^*∗∗*^*P* < 0.01 vs. control. (c) MC3T3-E1 cells were pretreated with different concentrations of paeoniflorin for 4 hours and then were incubated with 200 *μ*M DEX for 24 hours. Cell viability was measured by the CCK-8 assay.  ^*∗∗*^*P* < 0.01 vs. control;  ^*##*^*P* < 0.01 vs. DEX 200 *μ*M. Effect of paeoniflorin and 200 *μ*M DEX on alkaline phosphate (ALP) activity (d), alkaline phosphate staining (e), and Alizarin Red staining (f) in MC3T3-E1 cells. Data are presented as the mean ± SD.  ^*∗∗*^*P* < 0.01 vs. control;  ^*##*^*P* < 0.01 vs. DEX 200 *μ*M.

The influence of paeoniflorin and DEX on MC3T3-E1 cell viability was evaluated by CCK-8 assay to determine the apoptotic function of DEX as well as the protective function of paeoniflorin. As evident in Figure 1(a), the viability of MC3T3-E1 cells began decreasing upon exposure of cells to DEX at a concentration of 1 *μ*M. The inhibitory function was dose-related, with MC3T3-E1 manifesting a noticeable drop in viability at a DEX concentration of 200 *μ*M. In light of this observation, we used 200 *μ*M in our DEX model of reduced cell viability.

We also attempted to test the toxic influence of paeoniflorin on MC3T3-E1 cells. At less than 20 *μ*M, no obvious decrease was observed in MC3T3-E1 cell viability; however, paeoniflorin began to exert its toxic effects at 50 and 100 *μ*M (Figure 1(b)). To verify the protective function of paeoniflorin against DEX, we treated MC3T3-E1 cells with various concentrations of paeoniflorin prior to their exposure to 200 *μ*M DEX. CCK-8 assay indicated that 10 and 20 *μ*M paeoniflorin has the potential to alleviate the inhibitory effects of DEX on the proliferation of MC3T3-E1 cells (Figure 1(c)). ALP activity, ALP staining, and Alizarin Red staining were employed to validate the ability of paeoniflorin for stimulating osteogenic differentiation. As illustrated in Figures 1(d) and 1(e), DEX treatment resulted in a dramatic decrease in ALP activity and extent, whereas 10 and 20 *μ*M paeoniflorin subsequently promoted ALP expression and staining, 10 *μ*M in particular. Additional results, shown in Figure 1(f), suggested that paeoniflorin has the potential to stimulate the osteogenic mineralization of MC3T3-E1 cells that have been exposed to DEX. It is very much evident from the above findings that paeoniflorin can not only enhance the differentiation of MC3T3-E1 cells but also protect against the inhibitory effects of DEX.

### 3.2. Paeoniflorin Protects Preosteoblast MC3T3-E1 Cells from Apoptosis

**Figure 2:**
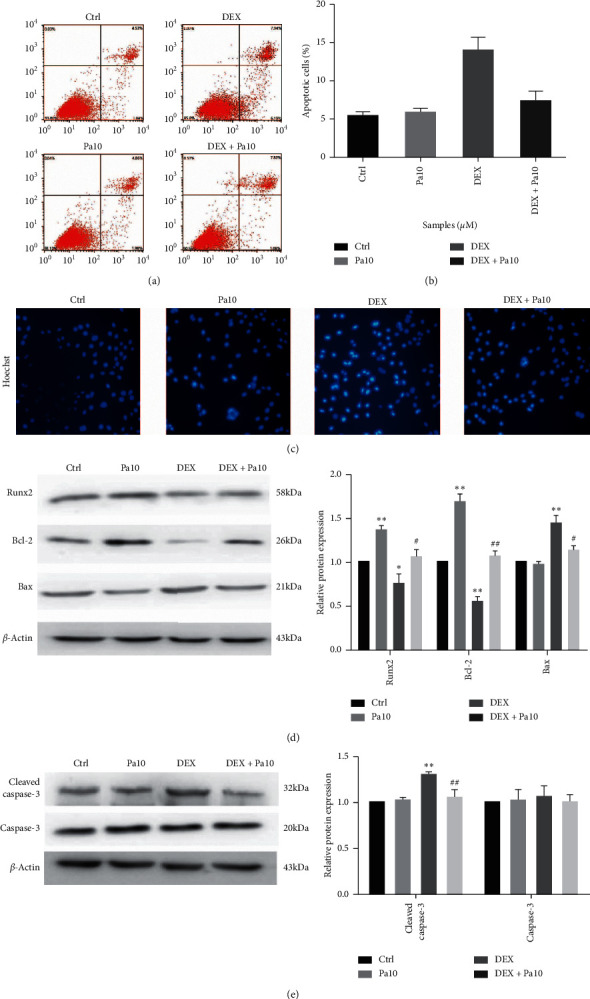
Effect of paeoniflorin on DEX-induced apoptosis in MC3T3-E1 cells. (a, b) The extent of apoptosis of MC3T3-E1 cells was detected by flow cytometry after Annexin V-FITC/PI double staining. The apoptotic rate was measured by flow cytometric analysis. Data are presented as the mean ± SD.  ^*∗∗*^*P* < 0.01 vs. control;  ^*#*^*P* < 0.05 vs. DEX. (c) MC3T3-E1 cells were stained by Hoechst to show the extent of apoptosis after interventions of DEX and paeoniflorin. (d) The expressions of osteogenic protein-Runx2 (runt-related transcription factor2) and apoptotic proteins such as Bcl-2 (B cell leukemia 2) and Bax (BCL2-associated X protein) were measured by western blot. ^*∗*^*P* < 0.05,  ^*∗∗*^*P* < 0.01 vs. control;  ^*#*^*P* < 0.05 vs. DEX;  ^*##*^*P* < 0.01 vs. DEX. (e) Protein levels of caspase-3 and cleaved caspase-3 were determined by western blot analysis, shown in quantitative analysis. Data are presented as the mean ± SD.  ^*∗∗*^*P* < 0.01 vs. control;  ^*#*^*P* < 0.05 vs. DEX.

Annexin V-FITC/propidium iodide flow cytometric analysis was carried out to test the degree of apoptosis in the intervention groups. In comparison to the control group, about 15% of MC3T3-E1 cells displayed an apoptotic appearance, whereas paeoniflorin pretreatment at 10 *μ*M only marginally alleviated DEX-induced apoptosis (Figures 2(a) and 2(b)). Meanwhile, Hoechst staining demonstrated that DEX causes an increased apoptosis, as indicated by dense nuclei and bright blue/indigo fluorescence; in contrast, paeoniflorin pretreatment reduced the count of apoptotic MC3T3-E1 cells (Figure 2(c)). Western blot was employed to determine the relative expression of proteins associated with osteogenic differentiation and apoptosis. As shown in Figure 2(d), Runx2 and Bcl-2 were found to reduce upon DEX treatment, with the elevated expression of Bax indicating stimulated apoptosis and inhibition of osteogenic differentiation. As expected, paeoniflorin was observed to reverse the inhibitory effects, meanwhile promoting the expression of Runx2 and Bcl-2 and the downregulation of Bax. Caspase-3 is considered to be a typical downstream effector of multiple apoptotic pathways. Cleaved caspase-3, the active form, has a key function in the apoptosis pathway. Therefore, we employed western blot to determine the degree of expression of cleaved and total caspase-3. Although the exposure of MC3T3-E1 cells to DEX resulted in a significant increase in the level of cleaved caspase-3, paeoniflorin pretreatment was subsequently able to attenuate the expression of cleaved caspase-3, as shown in Figure 2(e). The above findings demonstrated that DEX-induced apoptosis of MC3T3-E1 cells could be suppressed by paeoniflorin which additionally promotes osteoblastic differentiation.

### 3.3. Paeoniflorin Induces Autophagy in MC3T3-E1 Cells Exposed to DEX via AKT/mTOR Signaling Pathway Inhibition

**Figure 3:**
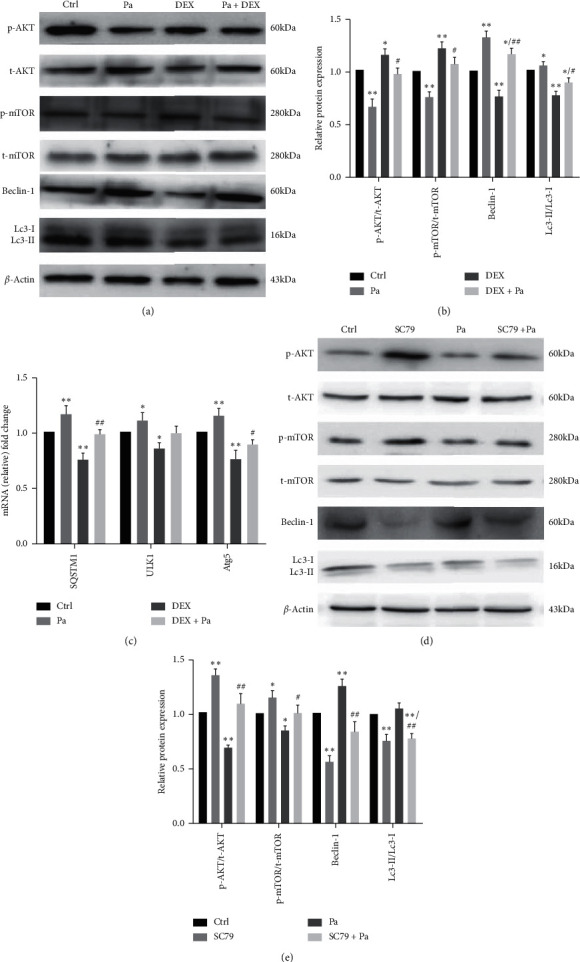
Regulations of AKT/mTOR pathway and autophagy by paeoniflorin after interventions of DEX in MC3T3-E1 cells. (a, b) Expressions of proteins involved in the AKT/mTOR pathway and autophagy were detected by western blot analysis. Data are presented as the mean ± SD.  ^*∗*^*P* < 0.05;  ^*∗∗*^*P* < 0.01 vs. control;  ^*#*^*P* < 0.05 vs. DEX;  ^*##*^*P* < 0.01 vs. DEX. (c) Expressions of representative autophagic mRNA as SQSTM1, ULK1, and Atg5 were determined by the measurements of RT-qRCR.  ^*∗*^*P* < 0.05,  ^*∗∗*^*P* < 0.01 vs. control;  ^*#*^*P* < 0.05 vs. DEX. (d, e) AKT agonist-SC79 was pretreated with MC3T3-E1 cells to identify the inhibition effect of AKT/mTOR pathway by paeoniflorin. Expression of related proteins was measured by western blot. Quantitated levels of protein were statistically evaluated.  ^*∗*^*P* < 0.05;  ^*∗∗*^*P* < 0.01 vs. control;  ^*#*^*P* < 0.05;  ^*##*^*P* < 0.01 vs. DEX.

In an attempt to further investigate the means by which paeoniflorin counteracts DEX, western blotting was performed to examine the changes in protein in cells exposed to paeoniflorin and DEX. The AKT/mTOR signaling pathway has a critical involvement in the autophagic activity, particularly after DEX treatment. Our results indicated that AKT and mTOR phosphorylation manifested a significant decline upon paeoniflorin treatment, with upregulation of beclin-1 (a key regulator of autophagosome formation) and LC3-II/LC3-I (a hallmark of autophagy activation) (Figures 3(a) and 3(b)), thus revealing that paeoniflorin pretreatment has the potential to induce autophagy by inhibition of the AKT/mTOR signaling pathway. Autophagy-related genes such as SQSTM1, ULK1, and Atg5 were analyzed using qRT-PCR (Figure 3(c)). DEX resulted in a dramatic decrease in their mRNA levels, whereas paeoniflorin reversed the inhibitory effect and promoted autophagic gene expression.

To investigate whether or not the AKT/mTOR signaling pathway is modulated by paeoniflorin, the AKT activator SC79 was administered to MC3T3-E1 cells treated with paeoniflorin, and later western blotting was carried out to test the levels of AKT and mTOR and their phosphorylated forms, as well as the typical markers of autophagy, such as beclin-1 and LC3-II/LC3-I. The results demonstrated that exposure to SC79 causes an increment in the expression of the AKT/mTOR signaling pathway and reversed the promotion of autophagy caused by paeoniflorin in MC3T3-E1 cells (Figures 3(d) and 3(e)). Taken together, paeoniflorin efficiently protects against DEX-induced apoptosis by inducing autophagy via the AKT/mTOR signaling pathway.

### 3.4. Paeoniflorin Promotes Osteogenic Differentiation and Autophagy in GIOP Mice

**Figure 4:**
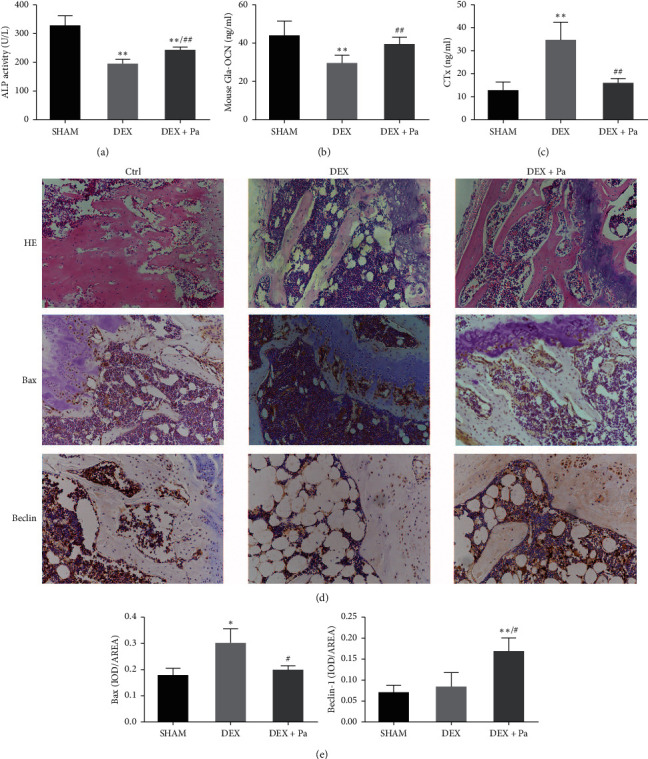
Paeoniflorin promotes the bone turnover marker in the serum of GIOP mice and regulates the apoptosis and autophagy of MC3T3-E1 in GIOP mice. (a–c) The serum levels of ALP, OCN, and CTx were detected by ELISA tests.  ^*∗∗*^*P* < 0.01 vs. sham;  ^*##*^*P* < 0.01 vs. DEX. (d) Effects of paeoniflorin on histological assessment of bone sections in GIOP mice (hematoxylin and eosin staining: magnification, x100). The areas stained red represent bone trabeculae. Immunostaining of Bax and Beclin-1 was captured at 200x magnification. (e) Bax and Beclin-1 staining was quantified by a bone histomorphometric analysis using Image-Pro Plus software. Columns represent mean ± SD from at least three independent experiments,  ^*∗*^*P* < 0.05;  ^*∗∗*^*P* < 0.01 vs. sham;  ^*#*^*P* < 0.05 vs. DEX.

To investigate the bone metabolism of GIOP mice after paeoniflorin pretreatment, ELISA was used to determine the concentrations of ALP, OCN, and CTX in the serum of GIOP mice. The results showed that DEX caused a decrease in the expression of the osteogenic markers ALP and OCN meanwhile increasing the expression of CTX, suggesting an obvious imbalance in bone formation and absorption (Figures 4(a)–4(c)). H&E staining of the epiphyseal growth plate in the distal part of the femur revealed significantly lower trabecular thickness in DEX-treated mice than in DEX + paeoniflorin treated mice (Figure 4(d)). Additional immunohistochemistry suggested that paeoniflorin blocked the increased expression of Bax and beclin-1 in the DEX + paeoniflorin group in comparison with the DEX group (Figures 4(d) and 4(e)). Therefore, as expected, paeoniflorin exerts an antiapoptotic influence and induces autophagy in the presence of DEX in GIOP mice.

## 4. Discussion

GC treatment is precisely a double-edged sword. While GCs have the potential to suppress inflammation, they can affect the balance between bone formation and bone resorption. Osteoporosis is characterized by a decline in bone information and bone mass and a simultaneous increase in bone resorption and bone loss, thereby leading to an increased risk of fracture [16]. Reduced osteoblast viability is the primary effect of excess GCs on bone metabolism, which can subsequently lead to a decrease in osteoblast numbers without affecting bone resorption [17]. As a consequence, the levels of ALP, a marker for early-stage osteogenic differentiation, and OCN, a marker for late-stage, are both found to decrease upon GC treatment. Our experiments produced quite similar results (Figure 4(a)). Furthermore, GCs depress the expression levels of collagen I and the osteogenic differentiation-related gene Runx2 [18]. Runx2 is an important marker of osteogenic differentiation that functions as a transcription factor for promoting osteogenic gene expression and regulating the expression of ALP, OCN, and collagen I. Results of the current study showed that Runx2 was decreased by DEX (Figure 2(d)), thus suggesting the impairment of bone formation activity by increased apoptosis in osteoblasts.

Osteoblasts are identified as bone-building cells of mesenchymal origin; they differentiate from mesenchymal progenitors, either directly or via an osteochondroprogenitor. The direct pathway is more or less typical for intramembranous ossification of the skull and clavicles, while the latter is a hallmark of endochondral ossification of the axial skeleton and limbs. At the level of preosteoblasts, however, the pathways converge and subsequently progress through 3 stages, namely, proliferation, matrix maturation, and mineralization. Osteoblasts, which are stellate cells populating small interconnecting passages within the bone matrix, may also divide into osteocytes. The primary molecular transition is the mesenchymal progenitors' contribution to orthostasis [19]. Decreased levels of osteoblasts and an increased osteoblast apoptosis have also been frequently reported in GIOP patients [20]. DEX induces apoptosis of MC3T3-E1 cells through activation of caspase-3, cleaved caspase-3 in particular, which precisely is a general downstream component of apoptotic signaling pathways [21]. Analysis of the expression levels of total and cleaved caspase-3 demonstrated that, whereas total caspase-3 remained unchanged, DEX stimulated the cleavage of caspase-3 and its function as an apoptosis effector (Figure 2(e)). Thus, the overall homeostasis of osteoblasts exposed to excess GCs is important to treat GIOP. There has been an increasing evidence that shows that a delicate interplay between autophagy and apoptosis is crucial for modulating osteoblastic cellular homeostasis [22]. Generally, an excess of GC can improve the Bax/Bcl-2 ratio and cause a decrease in the expression of autophagy-related genes such as beclin-1 and LC3-II, as shown in Figure 2(d) and 3(a). Bcl-2 interacts with beclin-1 and influences the switch between apoptosis and autophagy [23]. Thus, further exploration of GIOP progression needs to examine the relationship and crosstalk between apoptosis and autophagy. However, autophagosomes will discard cell proteins and organelles, when autophagy becomes excessive leading to death [24]. Therefore, the response to GC dosages determines the cellular status. High concentrations (>1 *μ*M) of DEX can continuously inhibit cell viability and accelerate apoptosis, but lower-dose DEX does not have an impact on viability [25]. At concentrations less than 5 *μ*M, we found no difference in the viability of MC3T3-E1 cells exposed to DEX (Figure 1(a)). Our results suggest that the clinical dose and period of GC treatments should be meticulously monitored and reduced if possible.

Autophagy starts with autophagosome formation and is essentially genetically controlled by autophagy-related gene (ATG) family members [22]. Isolation membranes, a marker of the autophagy process, can originate from the endoplasmic reticulum, mitochondria, and plasma membranes [26]. Subsequently, the isolation membranes adopt a spherical form and pass through phases involving sequential elongation and expansion, thereby acquiring a double membrane structure. During this period, LC3-II is a good indicator of autophagosome formation, having a fixed relationship with the number of autophagosomes. Upregulation of the expression of LC3-II predicts the accumulations of autophagosomes [27]. The results of our western blot analysis showed that DEX resulted in a reduction in the expression of LC3-II; this LC3-II expression was blocked by paeoniflorin pretreatment, which also induced autophagic activity (Figure 3(a)). At the same time, other autophagy-related genes were upregulated on qRT-PCR (Figure 3(c)). These findings demonstrate that paeoniflorin promotes autophagy.

Being a classical signal transduction pathway, the AKT/mTOR greatly affects the autophagic activity [28–30] and plays a vital role in the regulation of osteogenic differentiation [28]. In particular, mTOR is a negative regulator of autophagy initiation that can block the activation of Atg 1 kinase [22]. Therefore, the discovery of new drugs that have the potential to inhibit the AKT/mTOR pathway might be effective in rescuing osteoblasts from the apoptosis induced by DEX. Paeoniflorin has been previously reported for the inhibition of the AKT/mTOR pathway [29]. In this study, paeoniflorin pretreatment reduced the levels of phosphorylated AKT and mTOR (Figure 3(a)). Besides, SC79, an AKT activator, was used for investigating the effects of paeoniflorin on AKT/mTOR. Further, SC79 reversed the inhibitory influence of paeoniflorin on the AKT/mTOR pathway (Figure 3(d)), thus suggesting the protective effects of paeoniflorin against the apoptosis of osteoblasts by inducing autophagy through AKT/mTOR signaling pathway inhibition.

Paeoniflorin has been reported previously for increasing osteoblastogenesis [14]. It also effectively treats osteoporosis induced by high-carbohydrate, high-fat diet-associated hyperlipidemia [30]. However, the influence of paeoniflorin on GIOP remains rather unclear. ALP activity and Alizarin Red staining were found to be elevated following treatment with paeoniflorin *in vitro* which reflects the impact of paeoniflorin on osteogenic differentiation and mineralization (Figures 1(d)–1(f)). Furthermore, paeoniflorin reversed the bone resorption activity caused by DEX bone turnover markers and H&E staining of femur sections, thus promoting the growth of trabecular bone and the nearby metaphyseal endplate (Figures 4(a)–4(d)). Findings of the current study suggest that paeoniflorin alleviates the suppression of osteogenic differentiation and mineralization induced by DEX.

## Figures and Tables

**Table 1:** Sequences of target gene-specific primers used in RT-qPCR.

Gene	Forward	Reverse
ULK1	5'-AAGTTCGAGTTCTCTCGCAAG-3′	5'-CGATGTTTTCGTGCTTTAGTTCC-3′
SQSTM1	5'-AGGATGGGGACTTGGTTGC-3′	5'-TCACAGATCACATTGGGGTGC-3′
Atg5	5'-TGTGCTTCGAGATGTGTGGTT-3′	5'-GTCAAATAGCTGACTCTTGGCAA-3′

ULK1, unc-51-like autophagy activating kinase 1; SQSTM1, sequestosome 1; Atg5, autophagy-related 5.

## Data Availability

The data used to support the findings of this study are available from the corresponding author upon request.
